# Resident learning during a pandemic: Recommendations for training programs

**DOI:** 10.1017/cem.2020.435

**Published:** 2020-06-29

**Authors:** Garrick Mok, Nicholas Schouela, Lisa Thurgur, Michael Ho, Andrew K. Hall, Jaelyn Caudle, Hans Rosenberg, Shahbaz Syed

**Affiliations:** * Department of Emergency Medicine, University of Ottawa, Ottawa, ON; † Department of Emergency Medicine, Queen's University, Kingston, ON

**Keywords:** COVID-19, education, emergency medicine, pandemic

## Abstract

Resident education in emergency medicine (EM) relies upon a variety of teaching platforms and mediums, including real-life clinical scenarios, simulation, academic day (lectures, small group sessions), journal clubs, and teaching learners. However, the coronavirus disease 2019 (COVID-19) pandemic has disrupted teaching and learning, forcing programs to adapt to ensure residents can progress in their training.^1^ Suddenly, academic days cannot be held in person, emergency department (ED) volumes are dynamically changing, and the role of residents in ED procedures has been questioned. Furthermore, medical student rotations through the ED have been cancelled, decreasing resident exposure to undergraduate teaching. These changes to resident education threaten resident wellness and will have downstream effects on training and personal professional development. In response, programs must develop strategies to ensure that residents continue receiving high-quality training in a safe learning environment. In this review, we will cover recommended strategies put forth by two large EM programs in Ontario (Table 1).

## RÉSUMÉ

La formation des résidents en médecine d’urgence (MU) dépend de la diversité des moyens et des formules d’enseignement, notamment des scénarios cliniques réels, de la simulation, de la formation théorique (exposés magistraux, séances en petits groupes), des clubs de lecture et de l’enseignement par les apprenants. Toutefois, la pandémie de COVID‑19 est venue perturber l’enseignement et l’apprentissage, obligeant les responsables de programmes à s’adapter afin que les résidents soient en mesure de poursuivre leur formation1. Du jour au lendemain, les cours d’enseignement théorique ne pouvaient plus se tenir en personne, le nombre de patients examinés au service des urgences (SU) variait considérablement et le rôle des résidents dans les interventions pratiquées au SU a été remis en question. De plus, les stages en médecine au SU ont été annulés, d’où diminution du temps d’enseignement par les résidents aux étudiants de premier cycle. Tous ces changements encore en cours aujourd’hui mettent en péril le bien-être des résidents et se répercuteront plus tard sur la formation elle-même et sur le perfectionnement professionnel personnel. Aussi les responsables de programmes doivent-ils élaborer des stratégies qui permettent aux résidents de recevoir une formation de qualité dans un environnement d’apprentissage sûr. Seront exposées dans l’article les stratégies mises de l’avant dans deux programmes importants de MU en Ontario (tableau 1).

**Table 1.** Summary of teaching strategies during COVID-19

	Pre COVID-19	During COVID-19
Academic sessions	Core rounds, grand rounds, and journal clubs with an emphasis on active learning	Active learning can be improved with incorporation of a pause procedure, smaller virtual group sessions, “flipped-classroom” model, and weekly quizzes Moderator to assist with session planning and monitoring discussions Panel-style discussions to maintain engagement
Simulation	Primarily performed in simulation centre	Virtual simulation sessions to practise many aspects of resuscitation
	Barriers to implementation of in situ simulation	In situ simulation directed at COVID-19 management
On-shift learning	High volumes granting many opportunities for learning but may be a barrier to direct observation	Dynamic volumes allowing for increased direct observation by staff, question-, and case-based teaching opportunities
	Residents generally perform or assist in most resuscitation procedures and practise leadership skills	Residents empowered to partake in various aspects of resuscitation, with potential exclusion from high-risk procedures (e.g., intubation) Added focus on leadership roles and crisis resource management skills Use of audio devices to allow those not in the room to learn from the experience
	Senior residents work on improving departmental management and flow, which is made possible by high patient volumes	Staff can increase cognitive burden for senior residents in times of low volumes by reviewing their own cases with the resident More direct observation from staff Ask staff physician about medical nuances and practice variations for cases
Teaching	Residents have the opportunity to teach medical students and junior residents on-shift and in the classroom	Virtual teaching sessions for medical students allow residents to hone their educational skills and provide an important service to medical student learning Senior residents continue teaching junior residents on shift
Wellness	In-person wellness activities, such as ice cream rounds, hikes, and social events	Implementation of virtual events, such as ice cream rounds, yoga, virtual social evenings (trivia, drinks, board games)
	Frequent check-ins from program directors, staff physicians, and residents	Virtual check-ins from program directors, staff physicians, and residents

## VIRTUAL ACADEMIC SESSIONS

Academic sessions (case-based and didactic-style core rounds, journal club) have traditionally been held in person. With COVID-19, some programs have transitioned to host these virtually via videoconference software. Akin to traditional didactic teaching, virtual sessions are a higher yield when learners have an active role.^2^ Virtually, active learning can be incorporated by using a pause procedure, where presenters pause when asking questions to learners.^3,4^ This pause allows learners time to clarify and assimilate information, helping facilitate a group discussion.^4^ Virtually, longer pauses may be required for participants to unmute their microphone and/or type on a discussion board.

Another technique to facilitate active learning is splitting a larger group into smaller groups, which allows learners the opportunities to participate more readily.^5^ In small groups, lecturers can use various teaching styles, such as problem-based learning, case-based learning, and/or exam-style questions.^6–8^ Virtually, creating various “virtual classrooms” with pre-assigned resident groups can facilitate this. Having members from various residency years allows for greater discussion and teaching from senior to junior residents. Furthermore, a “flipped classroom” model, where learners are given objectives for pre-reading, allows for more in-depth discussions and knowledge application.^9^ Lastly, weekly quizzes are provided to stimulate individualized learning.

To minimize disengagement during virtual sessions, one recommendation is the use of a moderator to assist with session planning and monitoring discussions. Furthermore, restricting time dedicated to one lecturer and using panels of individuals may improve attendee attention and participation. For example, rounds may be delivered via panel-style presentations where individuals provide shorter targeted presentations to focus on key take-home.

## SIMULATION

Simulation has played an increasing role in medical education and provides opportunities to practice high-risk scenarios in a low-risk, high-fidelity environment.^10–12^ Although residents are unable to attend sessions at simulation centres due to COVID-19 restrictions, simulation continues to play an important role in resident education. Many departments have accelerated their in situ simulation programs in response to COVID-19 for protocol development and revision, provider training, and team-based training.^13^ Resident involvement has been variable, but our sites have embraced the opportunity to incorporate trainees, especially since many will be working independently on the front line in the near future.

Virtual simulation is another option for simulation-based training.^12^ In a virtual simulation, residents lead a case and practise various elements of crisis resource management (CRM) that are paramount to a successful resuscitation. Although residents are unable to practise procedures in this modality, the virtual exercise allows for the teaching of medical and CRM aspects of EM, providing a pathway for success in clinical settings. In this platform, we recommend that residents go through cases virtually with the simulation team, with an emphasis on verbalizing their mental model to team members. Afterwards, a debrief is held to highlight key learning points from each case.

## ON-SHIFT LEARNING

Canadian EM residency programs have transitioned to competency-based medical education (CBME).^14^ CBME changes the assessment of trainees to involve more direct observation, with an aim to provide trainees with better coaching and programs and better assessment data to ensure trainee progression towards competency.^15^ Direct observation yields important learner data to support the improvement of identified deficiencies.^15–17^ However, a busy ED may pose a barrier to obtaining direct observations.^18^ During the pandemic, dynamic ED volumes may result in an overall decrease in clinical opportunities for learners. This decrease in volume, however, allows more time for residents to obtain direct observations from staff physicians. This results in more opportunities for targeted feedback to improve history taking, discharge instructions, procedural skills, and more. Reduced volumes also give an opportunity to engage in extra case-based and question-based learning on shift. This allows for supplementation of the resident's learning and leads to more academically fulfilling shifts.

Another change with COVID-19 is the resident role during resuscitations and aerosol-generating procedures (e.g., intubation). It is critically important to balance learning objectives with learner safety.^1^ To mitigate some of the lost clinical opportunity, residents can be empowered to partake in all other aspects of resuscitations. For example, when a staff physician is intubating, residents can be tasked with leading the resuscitation, allowing them to hone their resuscitation and CRM skills. If the learner remains outside of the room, a technique to maximize learning is to walk through the management plan with the staff physician, then actively watch the resuscitation. An audio device is helpful to listen to how the staff is managing the resuscitation. Including a debrief is important to address any knowledge gaps or management questions.

Furthermore, reductions in patient volumes can negatively affect senior residents’ abilities to develop department management, flow, and leadership skills while on shift. Some techniques to mitigate this include having the senior learner manage a larger percentage of the department volume, while the staff takes on a direct observational role. Staff can also “review” their patients with the senior resident to improve the resident's ability to handle cognitive burden, maintain situational awareness, and improve leadership skills. Reviewing cases also allows for a higher-level discussion around medical nuance and practice. It is important to highlight that, for these techniques to be effective, the staff needs to take an active role in providing feedback to the “independently functioning resident.”

For learners in the final year of their training, the examination deferral has influenced their approach to the last few months of residency – many are now focusing on the transition-to-practise component. In particular, absorbing as much tacit knowledge as possible from staff is the highest yield learning. Indeed, residents are seeking to find positive outcomes in this difficult time, focusing on the unique experiences, and growing as physicians in the process.^19^

## LEARNING TO TEACH

The transition from a learner to that of a teacher helps residents become experts in EM. Previously, the transition to teaching occurred naturally when senior residents supervised junior learners. However, with COVID-19, medical students were removed from clinical rotations. To help residents continue improving their teaching skills, one recommendation is to have senior residents teach didactic sessions covering core EM topics to clerkship students. These sessions help improve the skills of our clerkship students and ensures that residents are able to take the next step in their training. With the anticipated return of medical students in the ED, we have an opportunity to think of new ways to integrate them. Some recommended strategies include pairing trainees with senior residents, allowing medical students to learn and our residents to teach. Lastly, although medical students are not rotating through the ED, senior residents continue to have opportunities to supervise junior residents. Dynamic patient volumes may provide increased opportunity for senior residents to provide core and case-based teaching to junior learners.

## RESIDENT WELLNESS

Focused strategies to ensure resident wellness during the pandemic is paramount. Residents cannot engage in meaningful learning if their basic needs as individuals are not met. In response, programs should immediately engage in a series of efforts to mitigate threats to resident safety, perceived utility, and personal well-being.^20^ Some options include frequent team huddles in the ED, frequent check-ins with mentors and program directors, assignment of a near-peer wellness buddy, ice-cream rounds, and virtual social events.^21^ By continuing to prioritize wellness, residents can continue to thrive, despite this disruption to their medical and personal lives.

## CONCLUSION

**Figure 1.**
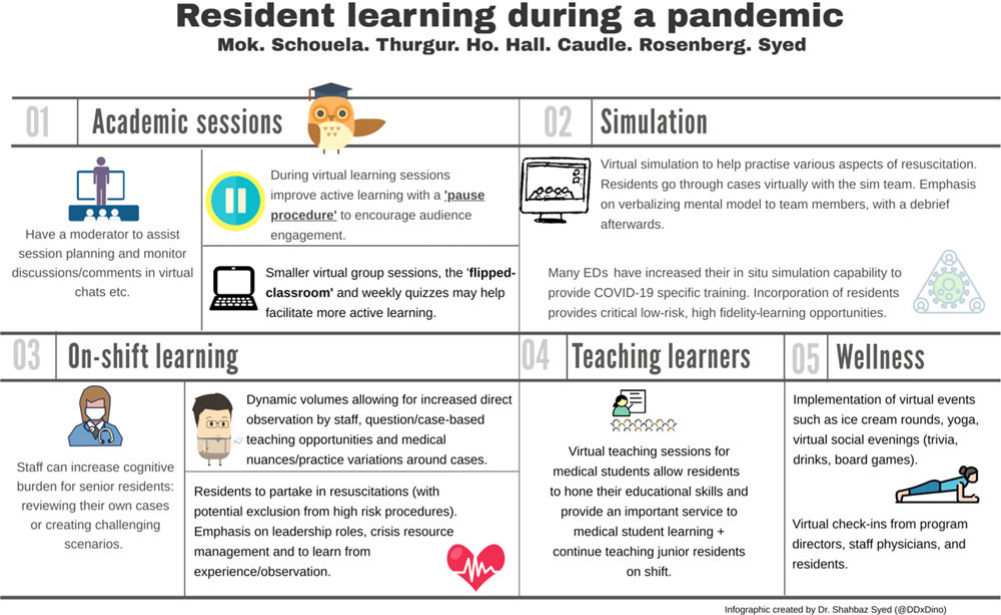
Learning strategies during a pandemic.

COVID-19 has forced programs to rethink educational strategies (Figure 1). Despite this, programs can find the silver-lining and embark on innovative ideas to improve resident education. With virtual academic sessions, some suggestions to help improve the sessions include adopting a flipped classroom model, using a pause procedure, and breaking a larger group to smaller group sessions. Virtual and in situ simulation offers opportunities to work on CRM of high-fidelity situations in a low-risk environment. On shift, learners should focus on obtaining direct observations, partaking in aspects of leading resuscitations, and discussing nuances and practice variations surrounding cases. As residents progress throughout their training, teaching learners plays an important role in the transition to becoming an emergency physician, and this should continue both on-shift and virtually. Overall, these suggestions can help residents continue to grow during this pandemic.
